# Genome‐Wide Cross‐Trait Analysis Dissects the Shared Genetic Architecture Between Type 2 Diabetes Mellitus and Metabolic Dysfunction–Associated Steatotic Liver Disease

**DOI:** 10.1155/humu/9992644

**Published:** 2026-04-09

**Authors:** Zijun Zhu, Hailong Li, Xin Wang, Xinyu Chen, Liang Cheng

**Affiliations:** ^1^ College of Bioinformatics Science and Technology, Harbin Medical University, Harbin, Heilongjiang, China, hrbmu.edu.cn; ^2^ NHC Key Laboratory of Molecular Probe and Targeted Diagnosis and Therapy, Harbin Medical University, Harbin, Heilongjiang, China, hrbmu.edu.cn; ^3^ State Key Laboratory of Frigid Zone Cardiovascular Diseases (SKLFZCD), Harbin Medical University, Harbin, Heilongjiang, China, hrbmu.edu.cn

**Keywords:** genome-wide association studies, nonalcoholic fatty liver disease, shared genetic architecture, Type 2 diabetes mellitus

## Abstract

The observational studies confirmed the high prevalence of metabolic dysfunction–associated steatotic liver disease (MASLD) in patients with Type 2 diabetes mellitus (T2DM), but whether this reflects a shared genetic etiology and exists underlying causal relationships remains unknown. Here, we utilized the largest scale cross‐trait analysis from genome‐wide association studies (GWASs) to investigate the shared genetic architecture and found a significant genetic correlation between T2DM and MASLD. Subsequently, we identified 581 shared risk single‐nucleotide polymorphisms (SNPs) and observed consistent patterns of tissue‐specific heritability enrichment in embryonic stem cells, stomach, kidney, large and small intestine, and pancreas. Of the six highly shared risk SNPs (rs7203132, rs11642015, rs58542926, rs6857, rs10404726, and rs738408), we further systematically performed regional and functional analysis. Using Mendelian randomization (MR), we discovered significant evidence for a positive causal effect with no reverse causality of T2DM on MASLD and further explained what causes causality to occur. Finally, we used an orthogonal strategy to provide genetic evidence, highlighting 11 possible comorbidity targets, most of which are located on Chromosomes 19 or 22 with five on 19p13.11, such as NCAN, MAU2, GATAD2A, TM6SF2, and GMIP. Our study sheds insights into the informed biology of comorbidity and reveals their shared genetic factors and potential drug targets.

## 1. Introduction

T2DM is characterized by dysregulation of carbohydrate, lipid, and protein metabolism, resulting from impaired insulin secretion, insulin resistance, or a combination of both [1]. MASLD is a disorder characterized by excessive accumulation of fat in hepatocytes and has emerged as the most prevalent liver disease globally [2]. T2DM and MASLD often coexist and synergistically elevate the risk of adverse clinical outcomes. Consequently, there is a pressing need to advance genetic research to elucidate the relationship between T2DM and MASLD and dissect the underlying biological mechanisms to alleviate the substantial global health burden imposed by these interconnected diseases.

Evidence supporting the reciprocal comorbidity of T2DM and MASLD has accumulated in recent years: A recent systematic review and meta‐analysis [3], encompassing 156 eligible studies and involving a pooled analysis of 1,832,125 patients, revealed a heightened prevalence of MASLD among individuals with T2DM (95% CI: 61.79%–68.15%, *I* ^2^ = 99.90*%*). Large‐scale GWASs targeting T2DM and MASLD have also identified hundreds of variants associated with these disorders. For instance, shared risk loci with genes—such as TM6SF2 [4, 5] and PNPLA3 [4, 5]—have been identified. While mounting evidence indicates a common genetic basis between the two conditions, the relational degree and shared genetic architecture remain undetermined [6]. Addressing these questions could help to gain a deeper understanding of the biological mechanisms underlying comorbid T2DM and MASLD and improve understanding of the genetic relationship between them, leading to safer and more effective interventions for both diseases when they occur alone or together.

**Figure 1:**
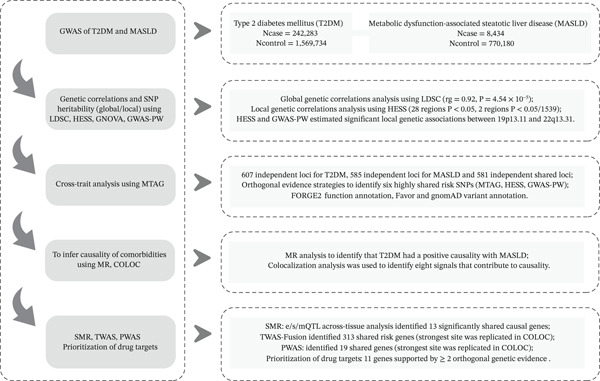
Study design.

Herein, we utilized large‐scale GWAS summary data to determine shared genetic risk loci and genetic structure between T2DM and MASLD. Next, Mendelian randomization (MR) was used to investigate evidence of a causal relationship. Following this, multiomics data combined with post‐GWAS analysis was employed to identify putative functional genes and facilitate the discovery of drug targets. Our investigation provides a deeper insight into the potential biological mechanisms underlying the comorbidity. The overall study design is shown in Figure 1.

## 2. Research Design and Methods

### 2.1. Study Samples

In this study, we acquired two recent largest scale GWAS summary statistics of European descent for MASLD and T2DM. The GWAS summary statistics data for T2DM was derived from the DIAGRAM, comprising 242,283 T2DM cases and 1,569,734 controls [7]. The GWAS summary statistics data for nonalcoholic fatty liver disease (NAFLD) (used as a proxy for MASLD) was derived from a meta‐analysis, which included 8434 cases and 770,180 controls [8]. Annovar (hg19_avsnp150, https://annovar.openbioinformatics.org/en/latest/) was used for variation annotation, and ambiguous SNPs were excluded to ensure the accuracy.

### 2.2. Heritability Estimation

Linkage disequilibrium score regression (LDSC) (https://github.com/bulik/ldsc), an effective method to estimate heritability from GWAS summary statistics [9], was used to estimate SNP‐based heritability (h^2^SNP) for T2DM and MASLD. Genetic covariance analyzer (GNOVA) (https://github.com/xtonyjiang/GNOVA) [10] was supplemented to estimate the h^2^SNP. Calculations were based on the reference files of the 1000 Genomes European ancestry data. The h^2^SNP was converted to liability‐scale heritability estimates using the following formula:$h_{l}^{2}=h_{o}^{2}\times K^{2}{\left(1-K\right)}^{2}/p\left(1-p\right)\times Z^{2}$, where *K* is the disease prevalence (T2DM: 22.51% [11]; MASLD: 32.40% [12]), *p* is the proportion of cases in the sample, and *Z* is the standard normal density at the liability threshold.

### 2.3. Global Genetic Correlation (*r*_*g*_)

We initially performed bivariate LDSC without constraining the intercept to estimate the *r*_*g*_s between T2DM and MASLD. The sensitivity analysis in the context of constrained LD score regression intercepts was employed to prevent population stratification and bias. Given that there is no sample overlap in our two traits, we set all single‐trait intercepts to 1 and all cross‐trait intercepts to 0. Subsequently, we used GNOVA to estimate genetic covariance based on all genetic variants shared between two GWAS summary statistics. *r*_*g*_s were then calculated based on variant heritability and genetic covariance.

### 2.4. Local *r*_*g*_s

Heritability Estimation from Summary Statistics (HESS) (https://huwenboshi.github.io/hess/) [13] is a method for estimating local SNP‐heritability and local genetic covariance (correlation) from GWAS summary association data. We used the 1000 Genomes European ancestry data and genome partition file for the specific chromosome as the reference provided by HESS. Local estimates were then calculated from the local h^2^SNP and local cross‐trait genetic covariance estimates.

The pairwise GWAS (GWAS‐PW) (https://github.com/joepickrell/gwas-pw) is a method to examine shared genomic regions by T2DM and MASLD. GWAS‐PW can provide estimates of the posterior probability for the locus shared by both traits (PPA3) or the locus associated with both traits but by distinct causal variants (PPA4). The threshold of PPA3/4 > 0.8 was used in our models.

### 2.5. Multitrait Analysis of GWAS

Multitrait analysis of GWAS (MTAG) (https://github.com/JonJala/mtag) [14] is a generalized cross‐trait analysis to account for sample overlap, imperfect *r*_*g*_, and genetic heterogeneity across different data sources for the same trait or different traits with a high *r*_*g*_. We used MTAG to identify risk SNPs for the combined T2DM and MASLD phenotypes. In the final MTAG analysis, after filtering out SNPs with MAF < 0.01, a total of 3,161,601 SNPs were retained.

### 2.6. Define Independent Risk Variant

Independent index loci were selected by the option “‐‐clump” using the PLINK v1.9 (https://www.cog-genomics.org/plink/) clumping method with a *p* value threshold at 5 × 10^−8^, clump *r* ^2^ at 0.01 with a window of 1 Mb. The reference files were from 1000 Genomes European ancestry data.

### 2.7. Tissue Enrichment

Functional element Overlap analysis of the Results of GWAS Experiments 2 (FORGE2) (https://forge2.altiusinstitute.org/) is performed to identify tissue‐ and cell type–specific signals for a given set of GWAS SNPs [15]. We used FORGE2 to evaluate evidence of tissue‐ and cell type–specific enrichment of signals shared by T2DM and MASLD.

### 2.8. MR Analysis

The best practice two‐sample MR inferences (https://mrcieu.github.io/TwoSampleMR/) were performed to evaluate the bidirectional causality between MASLD and T2DM, minimizing the potential bias from confounding and reverse causation. Genetic variants satisfying the following relevant assumptions can serve as instrumental variables (IVs): (i) strong correlation with exposure (*p* < 5 × 10^−8^), (ii) not in LD (*r* ^2^ < 0.001 within 10 Mb; the 1000 Genomes European ancestry data), and (iii) independent of outcome (not horizontally pleiotropic, *p*_MR−PRESSO_ > 0.05). These strict standards enable us to construct a robust aggregate of 360 IVs (*F* − statistics > 10). We used inverse‐variance weighted (IVW) [16], maximum likelihood [17], MR‐Egger [18], weighted median [19], and weighted mode methods to investigate putative causal relationships. We assessed heterogeneity across the MR estimates for each instrument in the IVW MR analyses with Cochran′s *Q* statistic and *I* ^2^ statistics (*p* < 0.05). The MR pleiotropy residual sum and outlier (MR‐PRESSO) and MR‐Egger regression indicate a measure of horizontal pleiotropy, and the MR‐PRESSO method was performed to identify outlier SNPs [20]. We conducted the leave‐one‐out (LOO) analysis to determine whether a single SNP was driving the link between exposure and outcome. As T2DM has overlapping samples with the selected MASLD, we applied a recent statistical method, MRlap [21], which calculates corrected causal estimates, accounting for sample overlap, winner′s curse, and weak instrument bias in MR. As post‐MR quality controls to avoid the deviation of IVs to MR analysis, we performed a directionality check of causal relationships by Steiger filtering [22].

### 2.9. Colocalization Analysis

Colocalization analysis (https://cran.r-project.org/web/packages/coloc/) primarily determines whether two or more phenotypes are driven by the same locus of causal variation in the same region. We screened for genome‐wide significant SNPs from traits, used LD clumping (*r* ^2^ < 0.001 within 1000‐kb windows) to look for independently correlated signals, and finally defined a ± 50‐kb region around them for colocalization analysis. Colocalization analyses were conducted by estimated regression coefficients (effect sizes) and standard errors. Here, we focused on the colocalizations when COLOC suggested a plausible posterior probability that both T2DM and MASLD are associated and share a single functional variant (PPH4 > 0.95). LocusZoom (http://locuszoom.org/) was used for visualizing the results in regional association plots.

### 2.10. Functional Annotation of Credible Variants

Functional annotation of variants–online resource (FAVOR v2.0) (https://favor.genohub.org/) is an open‐access variant functional annotation portal for whole genome/exome sequencing data [23]. We used FAVOR to functionally annotate six highly shared variants between T2DM and MASLD.

### 2.11. Summary Data–Based Mendelian Randomization (SMR)

The SMR (https://yanglab.westlake.edu.cn/software/smr/#SMR) is a method that integrates summary‐level data from GWAS with molecular quantitative trait locus (molQTL) data to identify genes whose expression levels are associated with a complex trait because of pleiotropy [24]. We utilized GWAS data for T2DM and MASLD, with eQTL, sQTL, and mQTL data from multiple tissues from FORGE2 (kidney cortex, liver, pancreas, small intestine terminal ileum, stomach, and whole blood) to apply SMR and HEIDI, which enabled us to investigate potential shared functional genes. The eQTL and sQTL datasets are sourced from GTEx v8 [25], and the mQTL data (whole blood) is obtained from McRae et al. [26]. SMR analysis results were required to pass the significance test (*p*_SMR < 0.05) and the HEIDI outlier test (*p*_HEIDI > 0.05).

### 2.12. Transcriptome‐Wide Association Study (TWAS)

TWAS has been commonly used to explore the relationship between gene expression and complex diseases and identify potential genes that influence the onset and progression of diseases [27]. We performed TWAS‐FUSION software (http://gusevlab.org/projects/fusion/) using GWAS summary statistics for T2DM and MASLD and gene expression data from multiple tissues from FORGE2 (kidney cortex, liver, pancreas, small intestine terminal ileum, stomach, and whole blood). The results prioritize genes with a significance threshold (*p*_TWAS < 0.05).

### 2.13. Proteome‐Wide Association Study (PWAS)

PWAS (http://nilanjanchatterjeelab.org/pwas/) is a method that assesses the association of genetically predicted protein levels with disease outcomes [28]. We used the reference files from the 1000 Genomes European ancestry data and protein weight files predicted from plasma proteins of European Americans for PWAS analysis. The results were specific to the statistically significant genes (*p*_PWAS < 0.05).

### 2.14. Genomics‐Driven Shared Druggable Targets Discovery

We prioritized genes that have at least two of the following biological lines of genetic evidence: (i) associated genes with highly shared loci identified jointly with MTAG, HESS, and GWAS‐PW; (ii) colocalization of priority causal genes; (iii) eQTL/sQTL/mQTL multi‐tissue colocalization by SMR&HEIDI; (iv) TWAS significant genes based on multi‐tissue eQTL data; and (v) PWAS significant genes based on plasma protein data. We then employed DrugBank (https://go.drugbank.com/) to identify drug targets and investigate associations with T2DM or MASLD based on corresponding drugs currently approved or in clinical trials and leveraged the Type 2 Diabetes Knowledge Portal (https://t2d.hugeamp.org/) to identify genes associated with either a single disease or comorbidities with at least moderate evidence range.

### 2.15. Functional Enrichment Analysis

Gene Ontology (GO) and Kyoto Encyclopedia of Genes and Genomes (KEGG) pathways analysis was performed. Three GO terms, biological process (BP), molecular function (MF), and cellular component (CC), were used to test if specific categories or pathways were enriched among the target genes.

## 3. Results

### 3.1. Global *r*_*g*_s Between MASLD and T2DM

To investigate whether and to what extent genetic factors share between T2DM and MASLD, we assessed the *r*_*g*_ using the bivariate LDSC analysis. The liability‐scale h^2^SNP estimates were 39.93% and 5.54% for T2DM and MASLD, respectively. From a global perspective, the positive genetic association seemed remarkably strong without constrained intercept (*r*_*g*_ = 0.92, *p* = 4.54 × 10^−5^, Table 1), suggesting a potential shared genetic basis and overlaps. The genetic covariance was calculated at approximately 0.0121 (Table 1), indicating little sample overlapping. In the case of constrained intercepts on the assumption of no population stratification, the genetic estimates are slightly weaker but remain significant (*r*_*g*_ = 0.80, *p* = 4.39 × 10^−17^, Table 1). In line with LDSC, GNOVA analysis showed a positive genetic association and consistent SNP heritability (Table 2 and Figure S1).

**Table 1:** Genetic correlation between T2DM and MASLD estimated using LDSC.

R_g_ (SE)	*p*value	Genetic covariance (SE)	T2DM	T2DM	MASLD	MASLD
			Lambda GC	Intercept	Lambda GC	Intercept
Without constrained intercept						
0.9185 (0.2252)	4.54e‐05	0.0121 (0.0010)	2.172	1.186 (0.0581)	1.0315	1.0137 (0.0084)
With constrained intercept of heritability						
0.7973 (0.0949)	4.39e‐17	0.015 (0.0009)	2.172	1	1.0315	1

Abbreviations: MASLD, metabolic dysfunction–associated steatotic liver disease; *r*_*g*_, genetic correlation; SE, standard error; T2DM, Type 2 diabetes mellitus.

**Table 2:** Heritability and genetic correlation between T2DM and MASLD estimated by various methods.

	Method	T2DM	MASLD
Heritability *h* ^2^	LDSC	0.1368 (39.93%)	0.0016 (5.54%)
Heritability *h* ^2^	GNOVA	0.1563 (45.62%)	0.0023 (7.97%)
Genetic correlation *r*_*g*_	LDSC	0.9185	
Genetic correlation *r*_*g*_	GNOVA	0.7884	

Note: *h* ^2^: SNP heritability.

Abbreviations: LDSC, linkage disequilibrium score; GNOVA, genetic covariance analyzer; *r*_*g*_, genetic correlation.

### 3.2. Local *r*_*g*_s Between MASLD and T2DM

We utilized HESS to estimate the local h^2^SNP and genetic association between MASLD and T2DM, identifying 28 suggestive significant correlations regions (*p* < 0.05, Table S1) and two significant correlations regions (19:18409862‐19877471 and 22:43714200‐44995308) after Bonferroni correction (*p* < 3.25 × 10^−5^, Figure 2 and Table 1). Additionally, we used GWAS‐PW to examine the shared genetic regions on the Chromosomes 8 (8:126410966‐128658961), 16 (16:53383142‐55903323), 17 (17:64800898‐67858387), 19 (19:18410369‐19876747, 19:19878263‐20902153, 19:16375173‐18409087, 19:44744870‐46101600), and 22 (22:43714255‐44993903) (Table S2). Consistent with previous studies [29, 30], significant local *r*_*g*_s were conservatively prioritized by complementary approaches—HESS and GWAS‐PW—on Chromosomes 19 and 22, suggesting that two regions (19p13.11 and 22q13.31) may be genetically associated with comorbidity.

Figure 2Local genetic correlation between T2DM and MASLD revealed by HESS. The plot shows the estimates of (a) local genetic correlation, (b) genetic covariance, and (c) SNP heritability between T2DM and MASLD. Significant local genetic correlation and genetic covariance estimates are highlighted in red and blue for even and odd chromosomes, respectively (*p* < 0.05/1,539).

**Figure:**
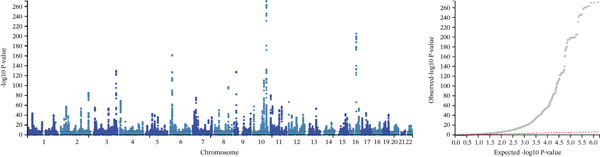
(a)

**Figure:**
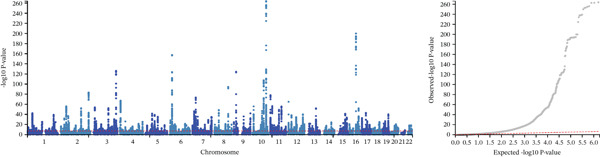
(b)

**Figure:**
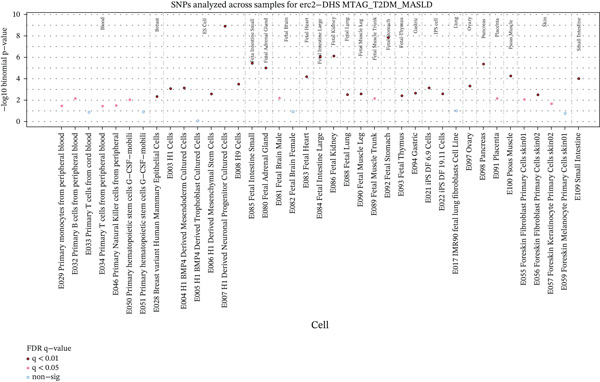
(c)

### 3.3. Cross‐Trait Analysis Identifies the Culprit SNPs

Given the strong genetic evidence between T2DM and MASLD, we conducted MTAG to improve the statistical power underlying the joint phenotypes. The cross‐trait analysis revealed 607 independent loci for T2DM (Figure S2a and Table S3) and 585 independent loci for MASLD (Figure S2b and Table S4), including 581 shared loci overall showing consistent association direction (*p* < 5 × 10^−8^, Table S5). The maxFDR values in MTAG analyses for T2DM (1.36 × 10^−5^) and MASLD (1.04 × 10^−5^) indicated that no genomic inflation and bias due to violation of MTAG assumptions is likely negligible. We then used FORGE2 to assess whether shared association signals enrich specific tissue types and discovered Bonferroni‐significant DNase I hotspots enrichment in the embryonic stem cells and fetal tissues, including stomach, kidney, and large and small intestine (Figure S2c and Table S6). The number of embryonic stem cells and blood was first‐ranked, exceeding even the more conventionally implicated multiple cell types and tissues.

### 3.4. Credible Variants and Functional Annotation

We used orthogonal evidence strategies—MTAG, HESS, and GWAS‐PW—to identify six highly credible shared risk SNPs (rs7203132, rs11642015, rs58542926, rs6857, rs10404726, and rs738408) mapped to six unique genes (Table 3 and Table S7). Among them, rs7203132 is a novel variant not associated with T2DM or MASLD, and rs11642015, rs6857, and rs10404726 are only associated with T2DM (known variants from the NHGRI‐EBI GWAS catalog). According to the Genome Aggregation Database v4.0.0 (gnomAD) [31], six highly shared risk SNPs showed nonlineage specificity. We then performed an integrated variant functional annotation approach using the FAVOR and RegulomeDB platforms for functional characterization. Of the six variants, four (rs11642015, rs10404726, rs58542926, and rs738408) were likely the QLTs for at least one tissue (Ranking = 1*f*), and rs58542926 was the more likely deleterious variant (CADD PHRED ≥ 12.37, Table S8).

**Table 3:** Highly shared genetic loci of T2DM and MASLD.

CHR	SNP	BP	A1	A2	MTAG_Pval_T2DM	MTAG_Pval_MASLD	Gene
16	rs7203132	53429775	A	G	6.81E‐13	1.36E‐12	LOC102723373 (dist = 11604); RBL2 (dist = 38608)
16	rs11642015	53802494	T	C	7.18E‐206	1.67E‐200	FTO
19	rs58542926	19379549	T	C	7.21E‐28	2.57E‐27	TM6SF2
19	rs6857	45392254	T	C	6.87E‐29	3.19E‐28	NECTIN2
19	rs10404726	18834514	T	C	1.03E‐15	2.55E‐15	CRTC1
22	rs738408	44324730	T	C	1.38E‐19	2.48E‐19	PNPLA3

*Note:* A1: effect allele; A2: other allele; MTAG_Pval_T2DM: *p* value of T2DM; MTAG_Pval_MASLD: *p* value of MASLD; GENE: nearest gene to the SNP.

The strongest association signal was localized on the alpha‐ketoglutarate–dependent dioxygenase (FTO) gene at locus 16q12.2, suggesting that FTO was the potential culprit gene. The index SNP rs11642015 was linked to T2DM (no association with MASLD) with a significance level of *p* = 6 × 10^−27^ and *p* = 1 × 10^−35^ in the previous GWASs [32, 33]. In the current MTAG T2DM GWAS, this variant demonstrated a significance level of *p* = 7.18 × 10^−206^, confirming the enhanced statistical power through the MTAG approach when boosting the effective sample size.

### 3.5. MR Determines Causal Evidence Between MASLD and T2DM

We conducted bidirectional MR to infer if the potential causal effect between T2DM and MASLD was consistent with pleiotropy or the presence of bilateral causality. Based on the three rigorous assumptions, the 360 IVs were selected after evaluation (Figure 3a and Tables 4 and S9). The IVW analysis, as the primary inference method, revealed an 11.18% increased risk for the occurrence of MASLD in T2DM patients (Figure 3b, OR = 1.1181, 95% CI: 1.0645–1.1744, *p* = 8.43 × 10^−6^), with no evidence of reverse causation using the Steiger test and reverse‐MR (*p* = 0.16). The consistency and statistical significance of the estimates from supplementary four bidirectional MR methods indicate that the causal conclusions based on these IVs are reliable (Figure S3a). No obvious pleiotropy or heterogeneity was detected in MR‐PRESSO (*p*_MR−PRESSO_ = 0.90, Figure S3b), Q statistics (*p*_IVW_Q_ = 0.90) and *I* ^2^ statistics (*I* ^2^ = 0*%*). LOO analysis identified no potential SNP outliers driving inconsistency or the effect driven by any single SNP (Figure 3c). Despite the MR‐Egger regression suggesting that the results may be affected by horizontal pleiotropy (*p*_MR−Egger_intercept_ = 7.40 × 10^−4^), this could be due to estimates from the MR‐Egger being likely to be affected by outlying and influential data points, leading to imprecision in the estimation, especially when the variant‐exposure associations are all similar in magnitude. To obtain a more precise estimate, we systematically refined the assumption to observe consistent causal estimates without heterogeneity or pleiotropy at a significance level of *p* = 2 × 10^−14^ (Figure 3d, OR = 1.0636, 95% CI: 1.0036–1.1273, *p* = 3.73 × 10^−2^). We observed positive LDSC intercepts, suggestive of sample overlap, but the MRlap estimates were consistent with the IVW estimates (beta = 0.0446, *p* = 1.39 × 10^−11^). No heterogeneity was observed (*p* = 4.44 × 10^−5^).

Figure 3MR determines causal evidence between MASLD and T2DM. (a) The workflow of MR analysis. (b) MR results show the effects of T2DM on MASLD, using inverse‐variance weighted and maximum likelihood methods. (c) The leave‐one‐out sensitivity analysis. (d) Causal estimation under different conditional assumptions.

**Figure:**
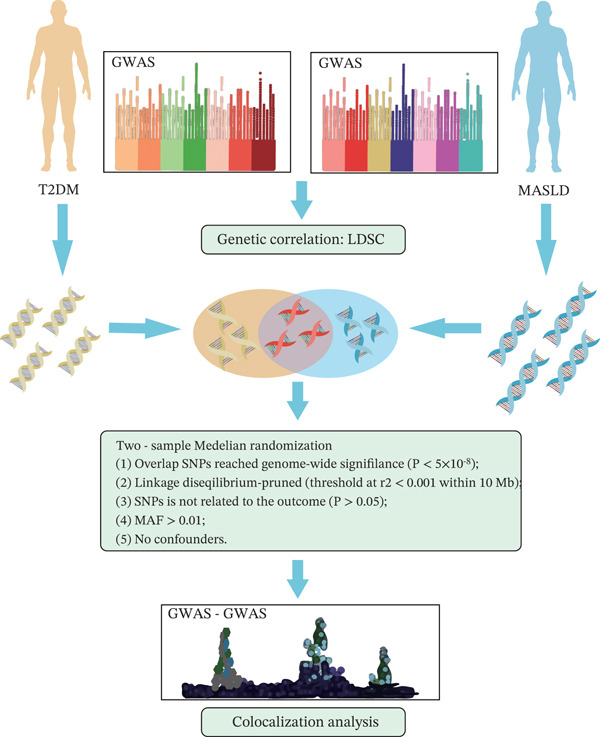
(a)

**Figure:**
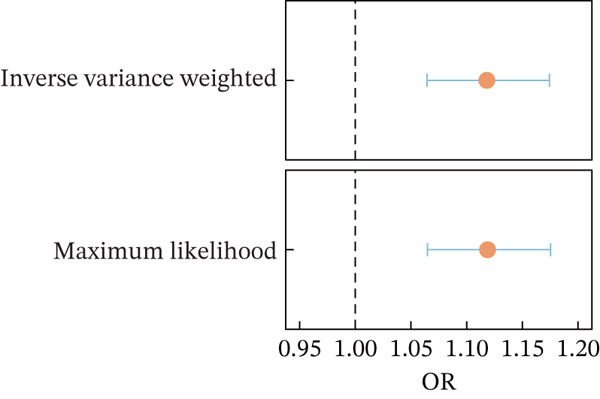
(b)

**Figure:**
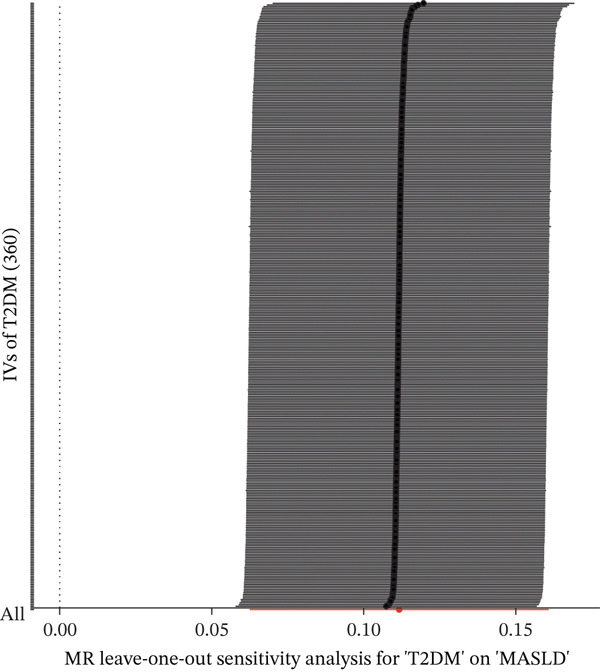
(c)

**Figure:**
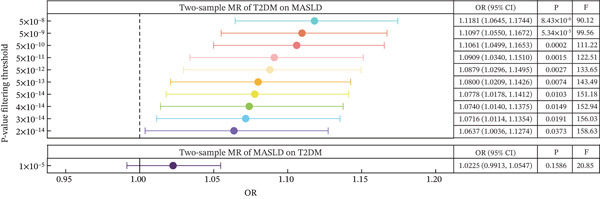
(d)

**Table 4:** Mendelian randomization (MR) inferences in evaluating the causal effects of T2DM on MASLD.

Exposure	Outcome	Assumption	IVs	IVW OR (95% CI) (*p* value)	Global test (*p*value)		Heterogeneity (*p*value)		
					*p* value of MR‐PRESSO	MR‐Egger intercept (*p* value)	IVW Q (P value)	MR‐Egger *Q* (*p* value)	*I* ^2^
T2DM	MASLD	5 × 10^−8^	360	1.118 (1.065–1.174) (8.43 × 10^−6^)	0.90	7.53 × 10^−3^ (7.40 × 10^−4^)	324.58 (0.90)	313.00 (0.96)	0
		2 × 10^−14^	145	1.064 (1.004–1.127) (3.73 × 10^−2^)	0.63	6.82 × 10^−3^ (8.64 × 10^−2^)	138.19 (0.62)	135.20 (0.67)	0

Abbreviations: 95% CI, 95% confidence intervals; GWAS, genome‐wide association study; IVs, instrumental variables; IVW, inverse‐variance weighted; MR‐PRESSO, Mendelian randomization pleiotropy residual sum and outlier.

To identify the genomic regions that cause causality, from colocalization analysis [34], we found several genomic regions (Table S10) spanning known susceptibility genes, such as MAU2 (rs73001065) [8], GATAD2A (rs3794991) [35], and TM6SF2 (rs58542926) on Chromosome 19, and SAMM50 (rs2294922) on Chromosome 22, with a high‐posterior probability (PPH4 > 0.95). Notably, the gene TM6SF2 with index variant rs58542926 overlapped for six highly shared risk SNPs obtained by MTAG analysis (Figure S4a,b).

### 3.6. Gene Expression/Alternative Splicing/DNA Methylation QTL Analysis Prioritizes Shared Causal Genes

To find the shared causal genes, thereby better analyzing the biological mechanism, we used SMR to identify putative functional genes by combining GWAS data with eQTL, sQTL, and mQTL data from multiple disease‐associated tissues. We identified 13 significantly shared causal genes (*p*_SMR < 0.05, *p*_HEIDI > 0.05). Among these, the NAGLU was identified through eQTL analysis, demonstrating significance for both T2DM (*p*_SMR = 0.03, *p*_HEIDI = 0.93, Table S11) and MASLD (*p*_SMR = 0.02, *p*_HEIDI = 0.99, Table S11). Additionally, mQTL analysis identified nine genes (Table S12), and sQTL analysis identified three genes (Table S13) with a shared cross‐tissue gene AMACR (liver, pancreas, kidney cortex, and stomach). Most genetic discoveries focused on whole blood (*N* = 11) rather than liver (*N* = 2), pancreas (*N* = 1), small intestine terminal ileum (*N* = 0), kidney cortex (*N* = 1), or stomach (*N* = 1).

### 3.7. TWAS Identify the Gene Direction Associated With Comorbidity

In TWAS analysis, we identified 313 risk genes significantly associated with T2DM and MASLD with one or more tissues (*p*_TWAS < 0.05, Table S14). MAU2, the prioritized susceptibility gene in colocalization, is located on Chromosome 19 and again identified by TWAS analysis as the strongest association signal shared by T2DM (*p*_TWAS = 9.29 × 10^−29^) and MASLD (*p*_TWAS = 3.00 × 10^−14^) in whole blood.

### 3.8. Mapping the Effects of Plasma Proteome on Risk of Comorbidity

We conducted the PWAS with plasma protein data from individuals of European descent and identified 19 shared proteins (Table S15). Consistent with colocalization, NCAN is the top signal located on Chromosome 19 (*p*_PWAS_MASLD = 2.10 × 10^−11^, *p*_PWAS_T2DM = 6.42 × 10^−19^), indicating that Chromosome 19 may be closely related to the occurrence of comorbidities.

### 3.9. Prioritization of Drug Targets for Comorbidity

Using multiple orthogonal genetic evidence, we selected 347 candidate genes most likely causative for comorbidities. Of the 11 genes supported by ≥ 2 criteria (Figure S5 and Table S16), DrugBank and Type 2 Diabetes Knowledge Portal were used to identify drug targets and potential associations with a single phenotype or comorbidities. We found four genes as drug targets (NAGLU, CYP21A2, GGT1, and NCAN). With at least moderate levels of evidence, three genes are specific for T2DM (NAGLU, CYP21A2, and NCAN); the gene NCAN is associated with MASLD specifically, and five genes are associated with the comorbidity (PNPLA3, MAU2, GATAD2A, TM6SF2, and GMIP). Functional enrichment analysis on 11 genes revealed significant enrichment primarily in “metabolic pathways,” “lipid homeostasis,” and the “lysosomal lumen.”

## 4. Discussion

In this study, we revealed shared genetic architecture and causal relationships between T2DM and MASLD. Combining multiomics information, we obtained 11 highly shared pathogenic genes, five of which are located in the 19p13.11, providing new insights into the shared genetic architecture of these two complex disorders.

We used LDSC to estimate the global genetic association between T2DM and MASLD and validated it by GNOVA methods, both demonstrating a significant *r*_*g*_. Meanwhile, local genetic association analysis—HESS and GWAS‐PW—showed a highly shared genetic region on Chromosomes 19 and 22. In the subsequent postanalysis, for example, the top associated genes (MAU2 and NCAN) were located on Chromosome 19 by TWAS and PWAS. Chen et al. [29] have identified related variants and genes of MASLD on Chromosomes 19 (19:19379549, TM6SF2) and 22 (22:44324730, PNPLA3). Unlike their study, our findings focused on the role and potential contributors of variants and genes between T2DM and MASLD.

MTAG, which involves a joint association analysis of genetically correlated traits, has demonstrated utility in boosting statistical power for detecting novel susceptibility variants relative to conducting separate GWAS for the individual traits tested [14]. In our study, MTAG was used to identify both independent and shared loci of T2DM and MASLD. Moreover, six highly shared risk SNPs located on or near the genes FTO (rs11642015), TM6SF2 (rs58542926), NECTIN2 (rs6857), CRTC1 (rs10404726), PNPLA3 (rs738408), and RBL2 (rs7203132) that were associated with T2DM and MASLD [4, 29, 30, 36, 37], were obtained by combining multiple methods (MTAG, HESS, and GWAS‐PW). The results suggest that these SNPs may be involved in regulating the common pathways shared between T2DM and MASLD, thereby leading to the occurrence and development of comorbidities.

We used MR analysis to identify that T2DM had a positive causality with MASLD, and there was no evidence for a reverse causal effect [38]. Furthermore, our study refined the traditional MR analysis by applying a stepwise tightening strategy at the significance level and observed consistent causal estimates with no heterogeneity or pleiotropy. Subsequently, we used colocalization analysis to identify the region that caused the causal relationship and further determined genes on Chromosomes 19 and 22, such as MAU2 and TM6SF2, with high posterior probability. However, it is important to consider that in addition to T2DM potentially leading to MASLD, there is also a situation where MASLD could increase the risk of developing T2DM [6, 39, 40], depending on factors such as data selection, threshold settings, and other methodological differences. For instance, different sample sizes, population characteristics, or how diseases are classified could influence the strength and direction of the associations observed. Despite the statistical significance, our finding still needs to be interpreted with caution as we cannot exclude that this positive association may have occurred by chance. Therefore, although we observed a clear causal link between T2DM and MASLD in our analysis, the possibility of MASLD promoting T2DM remains plausible and requires further investigation with different data sets and threshold criteria to better understand this relationship.

A previous cross‐trait analysis used MAGMA analysis and reported 16 genes overlapping between MASLD and T2DM, including IGF2BP2, SLC22A3, ZMIZ1, TCF7L2, FTO, and NPC1 [41]. Although the earlier study identified FTO as a shared gene—a finding corroborated by our identification of the index SNP rs11642015 near FTO—our approach highlighted a distinct set of 11 highly shared risk genes, most of which were located on Chromosomes 19 and 22 (8/11), especially NCAN, MAU2, GATAD2A, TM6SF2, and GMIP in 19p13.11, reflecting a highly shared genetic region. This discrepancy likely stems from methodological differences: [38] focused on gene‐level association testing, whereas our strategy integrated regional *r*_*g*_, causal inference, and molecular QTL colocalization to pinpoint genes with stronger evidence of shared biological mechanisms and potential druggability. Furthermore, we highlighted known drug target genes such as NAGLU, CYP21A2, GGT1, and NCAN, and potential targets such as PNPLA3 and TM6SF2. PNPLA3 encodes a protein that mediates triacylglycerol hydrolysis in adipocytes and may be involved in the balance of energy usage/storage in adipocytes [42]. TM6SF2 can be involved in the regulation of lipid metabolic processes [43]. Other nondruggable genes, including MRC2, OLFM2, MAU2, GATAD2A, and GMIP, also have potential implications for T2DM and MASLD. For example, MRC2 encodes a member of the mannose receptor family of proteins, and expression of this gene may play a role in the tumorigenesis and metastasis of several malignancies, including breast cancer, gliomas, and metastatic bone disease [44]. Our study may provide valuable support for drug development and treatment of T2DM and MASLD comorbidities.

This research has several advantages. (1) Our study is the first to use large‐scale genetic data to explore the shared genetic architecture between T2DM and MASLD. (2) The MTAG approach adopted in this study addresses the inclusion of datasets with overlapping samples, thereby minimizing the potential inflation of test statistics caused by such biases. (3) Improved MR strategies provide evidence for more reliable causal inference. (4) Multiomics approaches allow us to identify potential functional genes and drug targets.

There are some limitations to our study. (1) Our study utilizes GWAS data labeled as “NAFLD.” We recognize the recent paradigm shift towards the term “MASLD,” which formally integrates cardiometabolic risk factors—including T2DM—into its diagnostic criteria. This nosological update reinforces the core pathophysiological link between dysregulated metabolism and steatotic liver disease that our genetic findings elucidate. Consequently, the participant cohort in the “NAFLD” GWAS we analyzed would largely fulfill the current criteria for MASLD. (2) Our study was limited to European ancestry, thus limiting the ability to parse the genetic structure of other lineages. Furthermore, the examination of data quality found that there may be some group stratification. (3) In the postanalysis to identify potential functional genes for comorbidities, we gave priority to significant tissue types identified by FORGE2, but tissue selection may lead to bias in results. (4) Our proteomic‐based work has focused on plasma. Further proteomic studies need to involve more relevant fluids and tissues.

In conclusion, our findings highlight a potential genetic association between T2DM and MASLD and provide new insights into the shared genetic structure and biological mechanisms among them. These findings may provide important directions for future treatment strategies and risk prediction.

**Nomenclature**
MASLD: metabolic dysfunction–associated steatotic liver disease
T2DM: Type 2 diabetes mellitus
GWASs: genome‐wide association studies
SNPs: single‐nucleotide polymorphisms
MR: Mendelian randomization
LDSC: linkage disequilibrium score regression
GNOVA: genetic covariance analyzer
HESS: Heritability Estimation from Summary Statistics
GWAS‐PW: The pairwise genome‐wide association studies
MTAG: multitrait analysis of genome‐wide association studies
FORGE2: functional element Overlap analysis of the Results of genome‐wide association studies Experiments 2
MR‐PRESSO: Mendelian randomization pleiotropy residual sum and outlier
LOO: leave‐one‐out
FAVOR: functional annotation of variants–online resource
molQTL: molecular quantitative trait locus
TWAS: transcriptome‐wide association study
PWAS: proteome‐wide association study
GO: Gene Ontology
KEGG: Kyoto Encyclopedia of Genes and Genomes
gnomAD: Genome Aggregation Database

## Author Contributions

All authors made significant contributions: Z.Z. and L.C. designed the research; H.L., X.W., and X.C. performed the research; and H.L. and Z.Z. wrote the paper. Z.Z., H.L., and X.W. contributed equally to this work.

## Funding

This study was supported by the Tou‐Yan Innovation Team Program of the Heilongjiang Province (2019‐15), National Natural Science Foundation of China (10.13039/501100001809) (62222104, 62172130), and Heilongjiang Postdoctoral Fund (LBH‐Q20030).

## Conflicts of Interest

The authors declare no conflicts of interest.

## General Statement

*Code Availability.* Publicly available software tools were used to perform genetic analyses and are referenced throughout the manuscript.

## Supporting Information

Additional supporting information can be found online in the Supporting Information section.

## Supporting information


**Supporting Information 1** Figure S1: Genetic correlations between T2DM and MASLD were estimated using LDSC and GNOVA. No constrained intercept: estimation of without constrained intercept using LDSC; constrained intercept: estimation of constrained intercept using LDSC; GNOVA: estimation of genetic covariance analyzer.

**Supporting Information 2** Figure S2: Manhattan plots and tissue or cell type–specific enrich results for T2DM and MASLD. (a) The plot shows 607 independent index variants from T2DM MTAG in the European ancestry population. On the right is the quantile–quantile (Q‐Q) plot for T2DM MTAG GWAS in the European ancestry population. (b) The plot shows 585 independent index variants from MASLD MTAG in the European ancestry population. On the right is the Q‐Q plot for MASLD MTAG GWAS in the European ancestry population. (c) The dot plot identified tissue or cell type‐specific signals by analyzing 581 independent exponential variants shared by T2DM and MASLD. Solid red dots indicate *p* < 0.01 after FDR adjustment; pink solid point indicates *p* < 0.05 after FDR adjustment; blue hollow points indicate no significance.

**Supporting Information 3** Figure S3: Pleiotropy and heterogeneity test of MR. (a) Scatter plot shows the causal effects and pleiotropy test, using the IVW, maximum likelihood, MR‐Egger, weighted median, and weighted mode. The slope in each line represents the causal estimate of exposure on the corresponding outcome per method. (b) The funnel plot shows the heterogeneity.

**Supporting Information 4** Figure S4: LocusZoom plot for TM6SF2. (a) LocusZoom plot for TM6SF2 in T2DM GWAS dataset. (b) LocusZoom plot for TM6SF2 in MASLD GWAS dataset.

**Supporting Information 5** Figure S5: Summary of 11 highly shared genes in T2DM and MASLD comorbidities. The figure above depicts genes shared by five lines of biological evidence, a total of 11 genes supported by at least two lines of evidence.

**Supporting Information 6** Table S1: Summary of local genetic covariance between T2DM and MASLD. Table S2: Shared‐risk areas for T2DM and MASLD obtained by GWAS‐PW analysis. Table S3: Summary of all 607 genome‐wide significant loci in T2DM. Table S4: Summary of all 585 genome‐wide significant loci in MASLD. Table S5: Summary of all 581 shared genome‐wide significant loci in T2DM and MASLD. Table S6: Results for tissue‐ or cell type–specific signal from FORGE2 analysis. Table S7: Highly shared‐risk loci identified jointly with MTAG, HESS, and GWAS‐PW. Table S8: Functional annotation of highly shared‐risk loci for T2DM and MASLD using FAVOR. Table S9: Three hundred and sixty instrumental variables (IVs) were selected after evaluation. Table S10: Colocalization analysis with a high posterior probability (PP4 > 0.95) of T2DM and MASLD. Table S11: The eQTL multi‐tissues colocalization analysis in SMR. Table S12: The mQTL multi‐tissues colocalization analysis in SMR. Table S13: The sQTL multi‐tissues colocalization analysis in SMR. Table S14: Transcriptome full association studies in multiple tissues identified 313 unique genes associated with shared risk of T2DM and MASLD. Table S15: Protome‐wide association study of plasma proteins. Table S16: Eleven highly shared gene results.

## Data Availability

The summary statistics of MASLD and T2DM are publicly available.
