# Supplementary figures and images for: Genome‐Wide Cross‐Trait Analysis Dissects the Shared Genetic Architecture Between Type 2 Diabetes Mellitus and Metabolic Dysfunction–Associated Steatotic Liver Disease
Source: Hum Mutat. 2026 Apr 9;2026:9992644. doi: 10.1155/humu/9992644 (PMC13062659; doi:10.1155/humu/9992644)

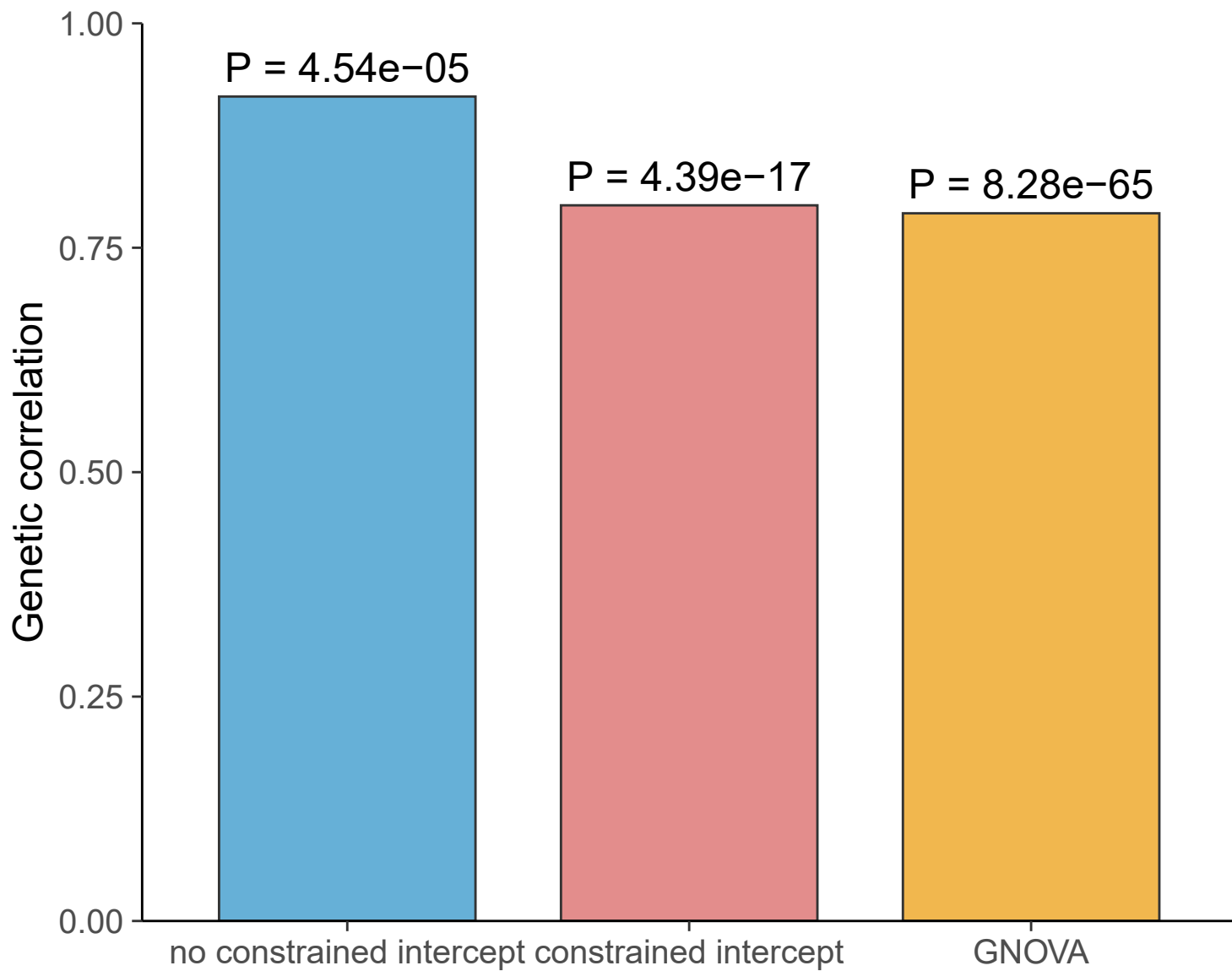

Supplement: Supplementary file 1 — Supporting Information 1 Figure S1: Genetic correlations between T2DM and MASLD were estimated using LDSC and GNOVA. No constrained intercept: estimation of without constrained intercept using LDSC; constrained intercept: estimation of constrained intercept using LDSC; GNOVA: estimation of genetic covariance analyzer. [file HUMU-2026-9992644-s001.pdf]

A

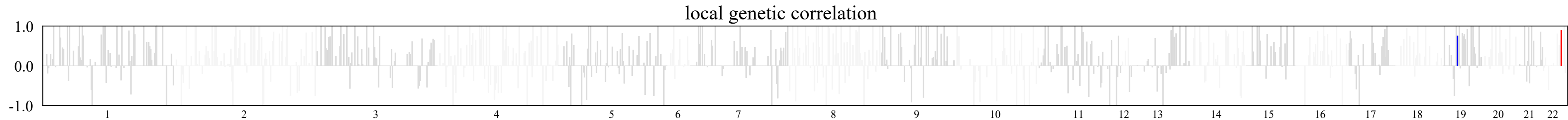

B

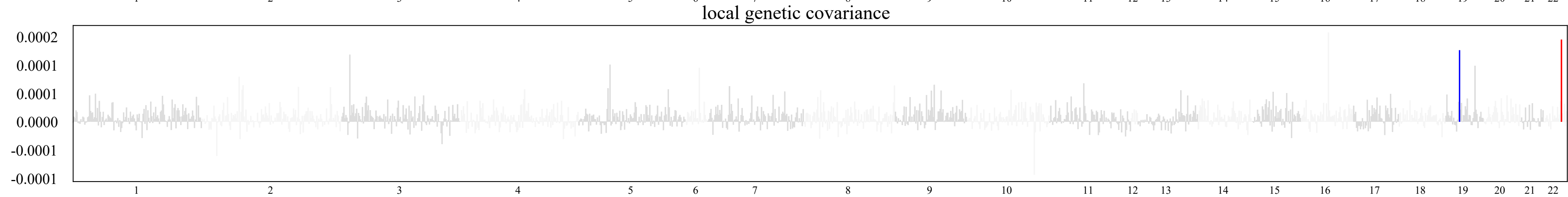

C

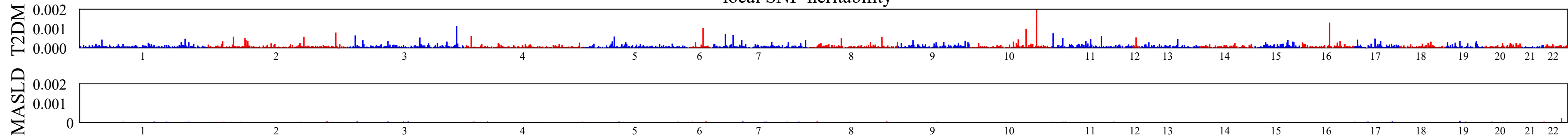

Supplement: Supplementary file 2 — Supporting Information 2 Figure S2: Manhattan plots and tissue or cell type–specific enrich results for T2DM and MASLD. (a) The plot shows 607 independent index variants from T2DM MTAG in the European ancestry population. On the right is the quantile–quantile (Q‐Q) plot for T2DM MTAG GWAS in the European ancestry population. (b) The plot shows 585 independent index variants from MASLD MTAG in the European ancestry population. On the right is the Q‐Q plot for MASLD MTAG GWAS in the European ancestry population. (c) The dot plot identified tissue or cell type‐specific signals by analyzing 581 independent exponential variants shared by T2DM and MASLD. Solid red dots indicate p < 0.01 after FDR adjustment; pink solid point indicates p < 0.05 after FDR adjustment; blue hollow points indicate no significance. [file HUMU-2026-9992644-s002.pdf]

A

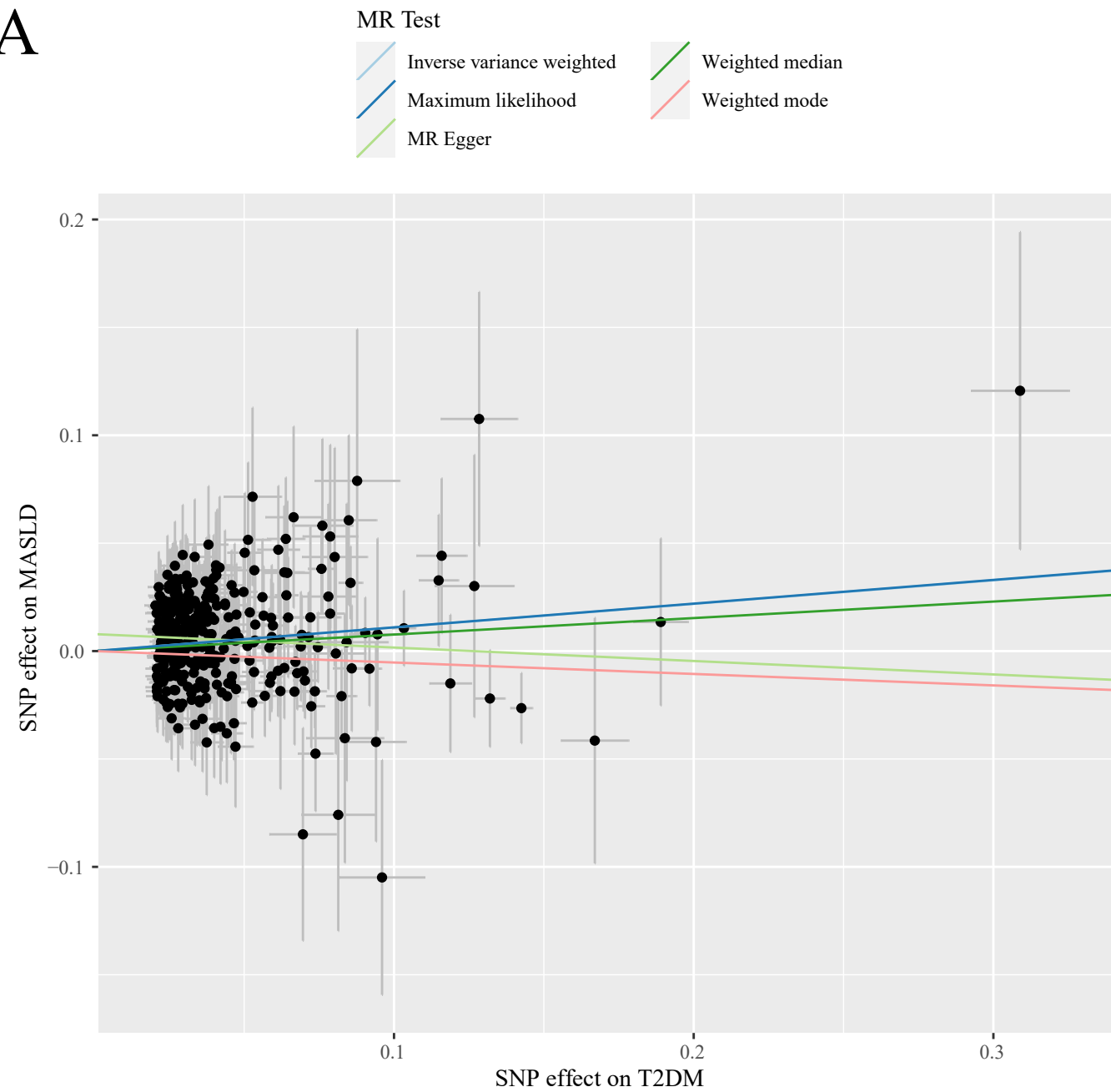

B

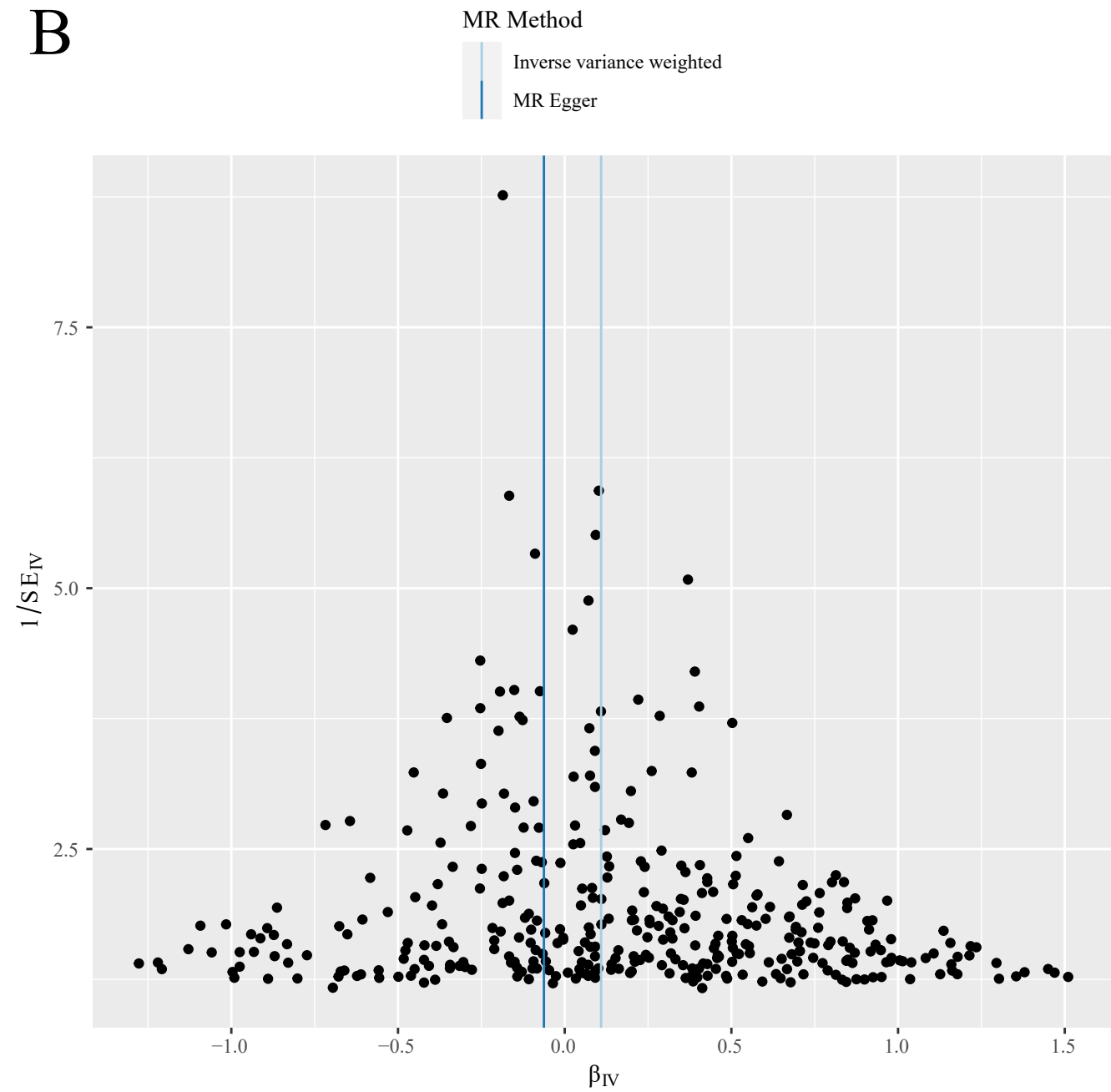

Supplement: Supplementary file 3 — Supporting Information 3 Figure S3: Pleiotropy and heterogeneity test of MR. (a) Scatter plot shows the causal effects and pleiotropy test, using the IVW, maximum likelihood, MR‐Egger, weighted median, and weighted mode. The slope in each line represents the causal estimate of exposure on the corresponding outcome per method. (b) The funnel plot shows the heterogeneity. [file HUMU-2026-9992644-s003.pdf]

A

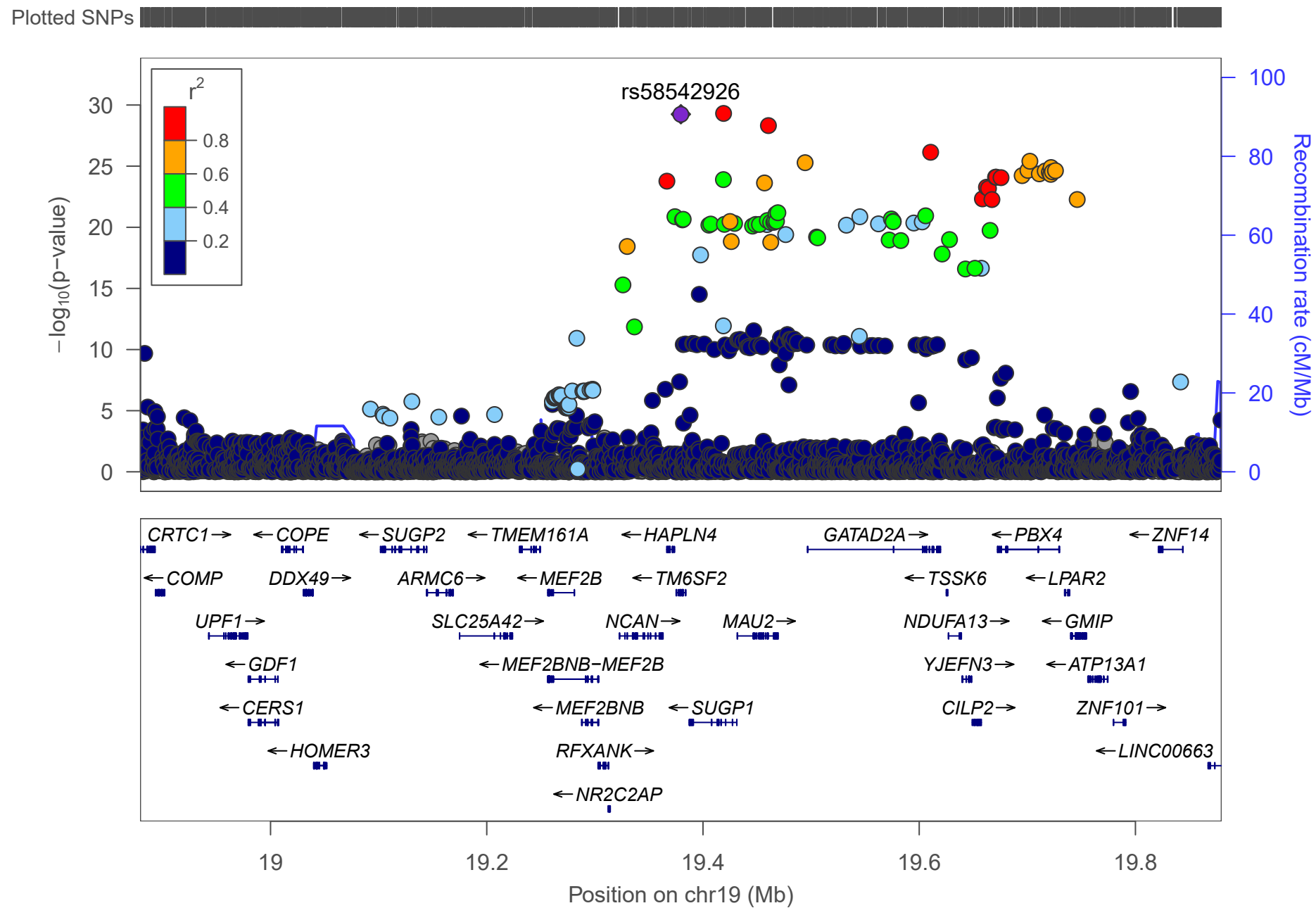

B

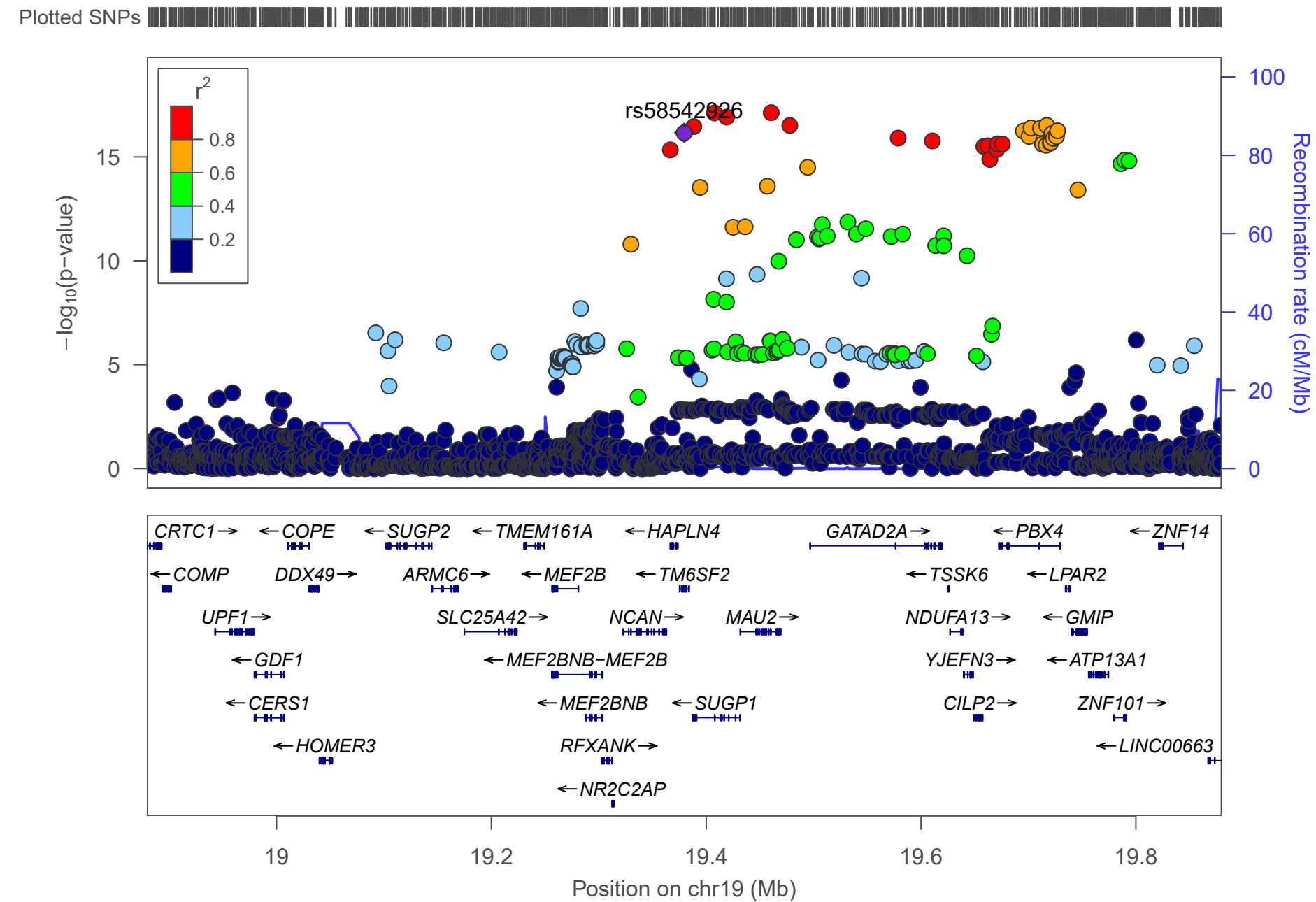

Supplement: Supplementary file 4 — Supporting Information 4 Figure S4: LocusZoom plot for TM6SF2. (a) LocusZoom plot for TM6SF2 in T2DM GWAS dataset. (b) LocusZoom plot for TM6SF2 in MASLD GWAS dataset. [file HUMU-2026-9992644-s004.pdf]

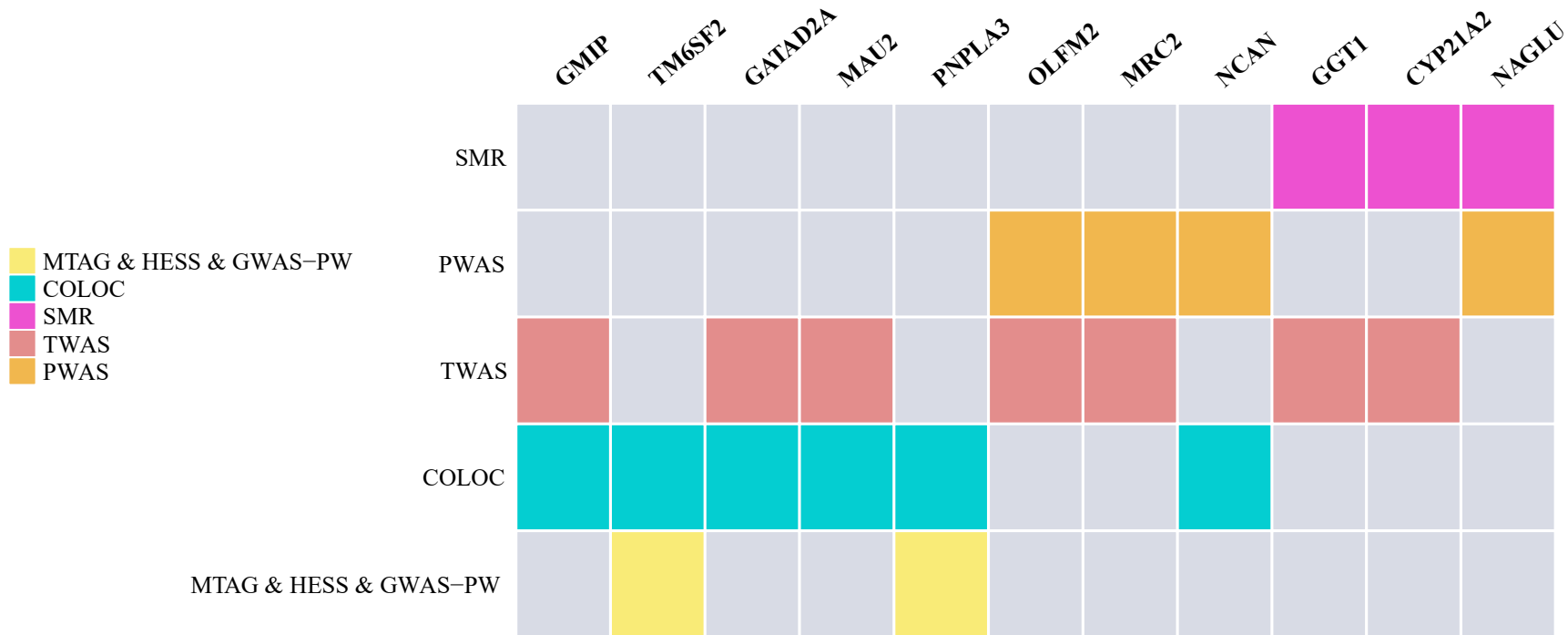

Supplement: Supplementary file 5 — Supporting Information 5 Figure S5: Summary of 11 highly shared genes in T2DM and MASLD comorbidities. The figure above depicts genes shared by five lines of biological evidence, a total of 11 genes supported by at least two lines of evidence. [file HUMU-2026-9992644-s005.pdf]
